# Protocol for a randomized controlled trial evaluating the effect of physical activity on delaying the progression of white matter changes on MRI in older adults with memory complaints and mild cognitive impairment: The AIBL Active trial

**DOI:** 10.1186/1471-244X-12-167

**Published:** 2012-10-11

**Authors:** Elizabeth V Cyarto, Nicola T Lautenschlager, Patricia M Desmond, David Ames, Cassandra Szoeke, Olivier Salvado, Matthew J Sharman, Kathryn A Ellis, Pramit M Phal, Colin L Masters, Christopher C Rowe, Ralph N Martins, Kay L Cox

**Affiliations:** 1 National Ageing Research Institute, Melbourne, Australia; 2 Department of Psychiatry, Academic Unit for Psychiatry of Old Age, St. Vincent’s Health, The University of Melbourne, Melbourne, Australia; 3 School of Psychiatry and Clinical Neurosciences and Western Australia Centre for Health & Ageing, University of Western Australia, Perth, Australia; 4 Department of Radiology, Royal Melbourne Hospital and The University of Melbourne, Melbourne, Australia; 5 Commonwealth Scientific and Industrial Research Organisation (CSIRO) Preventative Health Flagship, Melbourne, Australia; 6 CSIRO Preventative Health Flagship ICT, Royal Brisbane and Women's Hospital, Brisbane, Australia; 7 School of Exercise and Health Sciences, Edith Cowan University, Perth, Australia; 8 Mental Health Research Institute, The University of Melbourne, Melbourne, Australia; 9 Centre for Neuroscience, The University of Melbourne, Melbourne, Australia; 10 Department of Nuclear Medicine and Centre for PET, Austin Hospital, Melbourne, Australia; 11 School of Medical Sciences, Edith Cowan University, Perth, Australia; 12 School of Medicine and Pharmacology, University of Western Australia, Perth, Australia

## Abstract

**Background:**

Older adults free of dementia but with subjective memory complaints (SMC) or mild cognitive impairment (MCI) are considered at increased risk of cognitive decline. Vascular risk factors (VRF), including hypertension, heart disease, smoking, hypercholesterolemia and lack of physical activity (PA) have been identified as modifiable risk factors contributing to cognitive decline, and white matter hyperintensities (WMH) are associated with VRF, SMC and cognitive impairment. Findings from a growing number of clinical trials with older adults are providing strong evidence for the benefits of physical activity for maintaining cognitive function, but few studies are investigating these benefits in high-risk populations. The aim of AIBL Active is to determine whether a 24-month physical activity program can delay the progression of white matter changes on magnetic resonance imaging (MRI).

**Methods/design:**

This single-blind randomized controlled trial (RCT) is offered to 156 participants, aged 60 and older, in the Melbourne arm of the Australian Imaging Biomarkers and Lifestyle Flagship Study of Aging (AIBL). Participants must have SMC with or without MCI and at least one VRF. The PA intervention is a modification of the intervention previously trialed in older adults with SMC and MCI (Fitness for the Ageing Brain Study). It comprises 24 months of moderate, home-based PA (150 minutes per week) and a behavioral intervention package. The primary outcome measure will be change in WMH after 24 months on MRI. Cognition, quality of life, functional fitness, level of physical activity, plasma biomarkers for cerebrovascular disease and amyloid positron emission tomography (PET) imaging comprise secondary measures.

**Discussion:**

Currently, there is no effective pharmacological treatment available to delay cognitive decline and dementia in older adults at risk. Should our findings show that physical activity can slow down the progression of WMH, this RCT would provide an important proof of concept. Since imbedded in AIBL this RCT will also be able to investigate the interaction between vascular and Alzheimer's disease pathologies.

**Trial Registration:**

Australia New Zealand Clinical Trials Registry ACTRN12611000612910

## Background

The proportion of the global population aged over 60 years is expected to increase from 11% in 2009 to 22% by 2050 [1]. With increased longevity comes a greater risk of developing age-related diseases, including cognitive impairment and dementia. In 2010, the estimated number of people with dementia worldwide was 35.6 million with a projected increase to 115.4 million people by 2050 [2].

Older adults at risk of cognitive decline are those free of dementia but with subjective memory complaints (SMC) and mild cognitive impairment (MCI). SMC is defined as complaining about a deterioration of cognitive function without showing objective impairment on cognitive testing [3] and MCI additionally requires objective impairment on cognitive testing [4]. In the United States, approximately 22% of the population aged 71 years and older have cognitive impairment not reaching the dementia threshold [5].

White matter hyperintensities (WMH) on structural brain scans have been associated with cognitive impairment [6] and SMC in older adults [7] and have also been identified as a risk factor for Alzheimer’s disease (AD) in healthy older adults and MCI [8]. Further, WMH appear to be more common and extensive in individuals with vascular risk factors (VRF, eg hypertension, dyslipidemia, obesity, diabetes, smoking) and cerebrovascular disease (CVD) [9]. It has been hypothesized that CVD contributes to the increased risk for AD by damaging subcortical and cortical networks [10]. Brain white matter damage may be indicated by the appearance of WMH on T2-weighted images using magnetic resonance imaging (MRI) [8,11]. Thus, if the progression of white matter changes could be reduced via altering modifiable risk factors it might contribute to the prevention or delay of cognitive decline.

It is well established that physical activity (PA) is essential for maintaining physical abilities and independence in old age [12]. For optimal health benefits, older adults need at least 30 minutes of moderate-intensity aerobic activity on five days each week [13]. Aerobic activities are recommended to improve cardiovascular outcomes and modify VRF such as obesity [14], lipid profiles [15], blood pressure [16], glucose and insulin metabolism [17]. More recently, observational studies have demonstrated that PA is associated with a lower risk of cognitive decline and dementia [18,19]. Barnes and Yaffe [20] examined seven modifiable risk factors for AD and projected the effect of risk factor reduction on AD prevalence. They reviewed systematic reviews and meta-analyses and used relative risk estimates and prevalence data to calculate population attributable risk (PAR), which is the percentage of cases of AD attributable to each factor. Physical inactivity potentially contributed to 13% of all cases of AD worldwide (almost 4.3 million), which is the third largest proportion of AD cases after low education and smoking [20]. Further, Barnes and Yaffe [20] estimated that reducing inactivity by 10-25% could prevent between 380,000 to one million cases of AD globally.

To date, a few randomized controlled trials (RCT), literature reviews and meta-analyses have shown that PA has a positive effect on cognitive function for older adults with healthy cognition, SMC, MCI and dementia [21-26]. To our knowledge only one other PA study is currently underway that aims to reduce VRF in people at risk of dementia. Liu-Ambrose and colleagues [27] are conducting a RCT of six months of moderate-intensity aerobic exercise (walking) with 70 older adults with sub-cortical ischaemic vascular cognitive impairment. They will measure cognitive function, executive function, activities of daily living, physical function and blood biomarkers associated with metabolic syndrome and inflammation. However, no study has examined the brain white matter changes with PA.

Findings from animal research have generated several hypotheses to explain the cognition-enhancing effects of PA. These include neurogenesis [28,29], increased cerebral blood flow [30], reduced neuroinflammation [31], reduced brain beta-amyloid burden [32] and elevated levels of brain-derived neurotrophic factor (BDNF, [33]). To test these hypotheses, biological outcomes are starting to be measured in clinical studies involving PA. However, there have been mixed findings. For example, healthy older adults who undertook one year of moderate-intensity aerobic activity (three days per week) experienced a 2% increase in hippocampal volume as measured by MRI [34]. This increase was associated with an increase in serum BDNF and improved spatial memory. Baker and colleagues [22] measured fasting plasma levels of several AD-related biomarkers (including BDNF and beta-amyloids 40 and 42) in participants with MCI before and after six months of high-intensity aerobic exercise. Cognitive function improved, but, in contrast with Erickson et al. [34], the researchers reported a gender difference in plasma BDNF level. Compared with the control group, there was a trend towards increased circulating BDNF concentrations in men but reduced levels in women [22].

The main purpose and novel aspect of the present study is to conduct a methodologically rigorous RCT to determine whether PA can delay the progression of white matter changes, as measured by MRI, in community dwelling older adults with SMC and MCI who have at least one VRF. Secondary objectives are to investigate the effects of PA on cognition, well-being, physical function, plasma biomarker level for CVD and brain fibrillar beta-amyloid load measured with beta-amyloid positron emission tomography (PET) imaging. This paper describes the design of the AIBL Active study.

## Methods/design

### Study design

**Figure 1:**
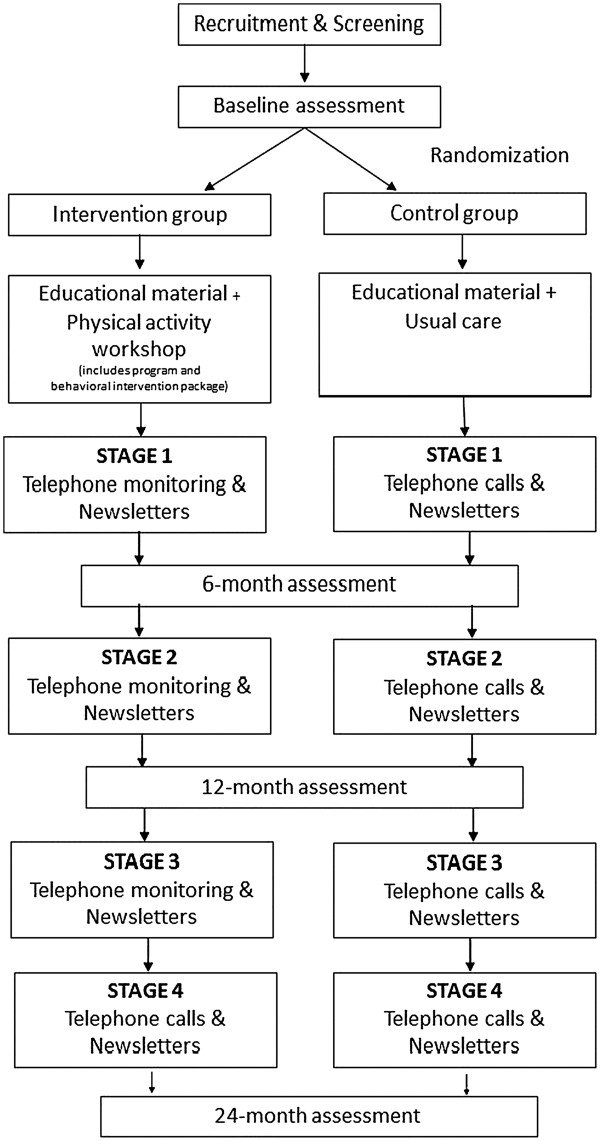
Study design.

AIBL Active is a single blind RCT (Figure 1). The CONSORT statement has been used as a framework for development of the methodology for this project.

### Participants

Community-dwelling older adults will be recruited in Melbourne, Australia. Participants are individuals with SMC or MCI with at least one VRF and who are also participants in the Australian Imaging, Biomarkers and Lifestyle (AIBL) flagship study [35]. AIBL is a multi-disciplinary prospective longitudinal study of ageing which follows 1112 older volunteers who live in Melbourne and Perth. The aim of this study is to identify the predictive value of biomarkers, cognitive variables and lifestyle factors for future progression to AD. AIBL participants belong to four diagnostic categories - normal cognition, SMC, MCI and Alzheimer’s disease. The AIBL study is ongoing, but to date 61% of MCI and 40% of SMC from both sites showed clinical progression on the Mini-Mental State Examination (MMSE) at 18 months follow-up. Of the 325 Melbournian participants with SMC and MCI, 214 (66%) have at least one VRF [35].

Participants will be eligible for enrolment in AIBL Active if they satisfy the following criteria: (i) aged 60 years or over at last birthday, (ii) diagnosis of SMC or MCI, (iii) community dwelling, (iv) presence of at least one VRF factor (such as obesity, hypertension, heart disease, type II diabetes, smoking, hypercholesterolemia and doing less than 150 min/week of moderate PA), (v) understands written and spoken English. Exclusion criteria include: (i) baseline Standardized Mini-Mental State Examination score (SMMSE) < 24 [36] or diagnosis of dementia, (ii) unable to have MRI scans, (iii) limited mobility (e.g. unable to walk or require a walking aid for balance), (vi) show evidence of pervasive depression, (v) current history of alcohol dependence, (vi) unstable or life-threatening medical condition, (vii) medical condition that contra-indicates PA, (viii) severe visual or hearing impairment, (ix) unable to attend the follow-up visits, or (x) participating in another RCT.

This study is funded by the National Health and Medical Research Council of Australia. Ethics approval has been obtained from the Melbourne Health Human Research Ethics Committee and the project complies with the Declaration of Helsinki.

### Recruitment and screening

Potential participants will be identified for inclusion and sent a letter of invitation by a member of the AIBL team. Each participant will be asked to contact the AIBL Active team by telephone and arrange a convenient time for a research assistant to check his/her eligibility (according to the study inclusion and exclusion criteria) using a screening protocol.

The 15-item Geriatric Depression Scale (GDS – 15) [37] is included as part of the phone screening to establish the presence of clinically relevant symptoms of depression, excluding potential participants with a score of 6 and higher. Participants will be asked to sign and return a release of medical information form to allow the study team to access their medical records and their physicians (and specialists if applicable) will be asked to consent to their patients’ involvement in the study. Medical records will be reviewed by a member of the research team who is an experienced Geriatrician to confirm the participants’ suitability for inclusion. Written informed consent will be obtained from participants prior to commencing the study.

### Assessments

Participants will be assessed at baseline and after six months, 12 months and 24 months (see Table 1 for an overview). There will be four components to the baseline and 24-month assessments comprising 1) cognitive measures; 2) physical function measures/physical activity assessment; 3) MRI; and 4) a fasting blood sample. From the 80 ml sample, lipid concentrations (total cholesterol, LDL-C, HDL-C, triglycerides), fasting insulin, glucose, homocysteine and hs-CRP will be measured. In addition, blood samples will be analyzed for the following biomarkers: IL-6, TNF-α, sICAM-1, sVCAM-1, sP-selectin, sE-selectin. At baseline only, participants will have a beta-amyloid PET scan. Participants will undergo only the first two components at their six- and 12-month visits (see Table 1 for the measures administered at each time point).

**Table 1:** Outline of the assessments and timelines of AIBL active

***Measure***	**Telephone Screen**	**Baseline**	**6 months**	**12 months**	**24 months**
Geriatric Depression Scale – 15 item	X				
Standardized Mini-Mental State Examination		X	X	X	X
Cambridge Contextual Reading Test		X			
Alzheimer’s disease Assessment Scale – Cognitive section		X	X	X	X
Clinical Dementia Rating Scale		X	X	X	X
Consortium to Establish a Registry for Alzheimer’s Disease battery		X			X
Wisconsin Card Sorting Test (WCST-64)		X		X	X
N-Back Task		X		X	X
Trails A & B/Trail Making Test		X		X	X
Behavior Rating Inventory of Executive Function – Adult Version		X		X	X
Short Form-36 version 2 (SF-36v2)		X	X	X	X
Hospital Anxiety and Depression		X	X	X	X
Memory Complaint Questionnaire		X		X	X
Everyday Competence Questionnaire		X	X	X	X
Resting blood pressure		X	X	X	X
Height, weight, body composition, girths		X	X	X	X
Step test		X	X	X	X
Sit-to-stand test		X	X	X	X
Grip strength		X	X	X	X
Timed Up and Go test		X	X	X	X
Six-minute walk		X	X	X	X
Community Healthy Activities Model Program for Seniors (CHAMPS) questionnaire		X	X	X	X
Stages of Change Instrument		X	X	X	X
Satisfaction with Life Scale		X	X	X	X
Self-Efficacy Questionnaire		X	X	X	X
Content and program evaluation			X	X	X
Blood sample collection		X			X
MRI scan		X			X
PET scan		X			

The X indicates at which point of the trial the respective assessments will take place.

With the exception of the SMMSE (because it is administered at the start of the visit to determine eligibility), the cognitive assessment will be administered by a neuropsychology research assistant who will remain blind to group allocation. The SMMSE and physical assessments will be conducted by a physical activity research assistant (PA RA). The SMMSE [36] is a modified version of the traditional Mini-Mental State Examination (MMSE) [38], which benefits from greater objectivity through more specific scoring examples as well as alternatives for repeated testing of the registration and delayed recall items. A score of less than 24 on the SMMSE excludes participants from AIBL Active. The PA RA will also collect demographic and health information via participant interview.

#### Measures administered by the neuropsychologist

The following 12 assessments comprise the cognitive battery. The Alzheimer’s disease Assessment Scale – Cognitive section (ADAS-cog) [39] consists of an 11-item battery of short neuropsychological tests and is widely used to monitor the progression of cognitive deficits in clinical trials. Here we will use a version incorporating a delayed verbal recall task.

The Cambridge Contextual Reading Test (CCRT) [40] includes the words of the National Adult Reading Test (NART), embedding them within appropriate semantic and syntactic contexts to provide a reliable measure of pre-morbid intelligence. The CCRT is only administered at baseline.

The Clinical Dementia Rating Scale (CDR) [41] is a widely used clinical staging instrument for dementia, useful for globally staging the level of impairment: 0 = No impairment, 0.5, 1, 2, and 3 indicate very mild, mild, moderate and severe dementia, based on a semi-structured interview.

The Consortium to Establish a Registry for Alzheimer’s Disease (CERAD) neuropsychological assessment battery [42] is sensitive to early cognitive impairment. The individual tests are: verbal fluency, Boston Naming Test, MMSE, word list and constructional praxis. It will be used to help determine whether participants have a classification of MCI at baseline.

The Wisconsin Card Sorting Test (WCST-64) [43] is a well- established measure of executive function and is more specifically a neuropsychological test of set-shifting, i.e. the ability to display flexibility in the face of changing schedules of reinforcement. The WCST-64 comprises two decks of 64 cards. The computerized version will be used, which allows more timely administration and instant scoring, while reducing the potential for administration and scoring errors.

The n-Back Task [44] is a measure of working memory. In the most typical variant of the n-Back Task, the participant is required to monitor a series of stimuli and to respond whenever a stimulus is presented that is the same as the one presented n trials previously, where n is a prespecified integer, usually 1, 2, or 3. The task requires on-line monitoring, updating, and manipulation of remembered information and is therefore assumed to place great demands on a number of key processes within working memory.

The Trails A & B/Trail Making Test (TMT) [45] provides information on visual search, scanning, speed of processing, mental flexibility, and executive functions. The TMT consists of two parts. TMT-A requires an individual to draw lines sequentially connecting 25 encircled numbers on a sheet of paper. Similar tasks are required for TMT-B except the person must alternate between numbers and letters.

The Behavior Rating Inventory of Executive Function – Adult Version (BRIEF-A) [46] is a standardized measure that captures views of an adult's executive functions or self-regulation in his or her everyday environment. The BRIEF-A is composed of 75 self-report items within nine non-overlapping theoretically and empirically derived clinical scales that measure various aspects of executive functioning.

The Short Form-36v2 [47] is a 36-item questionnaire that assesses the health and well-being of the participant across eight dimensions; physical functioning, social functioning, role limitations due to physical problems, role limitations due to emotional problems, mental health, energy/vitality, pain, and general health perception. Health change over the past year is also assessed.

The Hospital Anxiety and Depression Scale (HADS) [48] is a 14-item self-rating instrument designed to assess the presence and severity of anxiety and depressive symptoms in medical patients. The scale consists of separate seven-item subscales for depression and anxiety and can be used within hospital out-patient, primary-care, and community settings for all age groups.

The Memory Complaint Questionnaire (MAC-Q) [49] is a six-item scale of self-reported memory decline in which participants compare current memory ability with past performance for given situations. Scores range from 7 to 35 with higher scores reflecting perceived cognitive decline.

The Everyday Competence Questionnaire (ECQ) [50] comprises 17 items that evaluate a person’s ability to perform activities essential to independent living, such as housekeeping, leisure activities and mobility.

#### ***Measures administered by the PA RA***

The test battery for the physical assessment will include resting blood pressure, height, weight, body composition, waist and hip girths, step test for dynamic balance [51], Timed Up and Go (TUG) Test for mobility [52], five timed chair stands [53] for lower limb strength and maximum voluntary hand grip strength. All of these measures are described in the published protocol for the Fitness for the Ageing Brain Study II (FABS II) [54]. For cardiovascular endurance, participants will perform the 6-minute walk test [55]. Participants will be instructed to walk as fast as possible around a 20-metre measured course as many times as they can in six minutes. The maximum distance walked, heart rate and rate of perceived exertion [56] will be recorded.

Several questionnaires used in FABS II [54] will also be administered in AIBL Active, including the Community Healthy Activities Model Program for Seniors (CHAMPS) physical activity questionnaire [57], Stages of Change Instrument (SCI) [58], Self-Efficacy Questionnaire (SEQ) [59], and Satisfaction with Life Scale [60]. Participants will be provided with a pedometer (Digi-Walker SW-200, Yamax Inc., Tokyo, Japan) and asked to wear it for five weekdays and the weekend, following the baseline and other visits, to objectively measure their weekly PA. Participants will be shown how to wear and use the pedometer and how to complete the diary. Participants will be instructed to maintain their usual activity pattern during the monitoring period. Pedometers are recognised as objective and valid measures of PA in older adult populations [61].

#### Neuroimaging

##### MRI scan

All scanning will be performed on a Siemens (Erlangen, Germany) 3T Tim Trio Scanner using a 12-channel phased array coil. High resolution data sets will be acquired to allow accurate evaluation of brain atrophy and white matter disease. Seven MRI sequences will be acquired: 1) Sagittal T1 weighted Magnetization Prepared Rapid Gradient Echo (MPRAGE) MRI; isotropic 1mm voxel (TR = 1900ms, TE = 2.13ms, flip angle = 9°, TI = 900ms); 2) Sagittal 3D Fluid Attenuated Inversion Recovery (FLAIR); isotropic 1mm voxel (TR = 5000ms, TE = 355ms, flip angle = 120°, TI = 1800ms); 3) Axial T2 Turbo Spin Echo; 36 slices, 3mm thickness, 0mm gap, in plane resolution of 0.9x0.9 mm (TR = 3000ms, TE = 98ms); 4) Resting state imaging: 7min 35sec acquisition; Blood Oxygenation Level Dependent (BOLD) contrast, single-shot, T2*-weighted, gradient-echo planar imaging (EPI) sequence; 34-slice acquisition (TR = 2500 ms, TE = 30ms, flip angle = 90°, voxel size = 3.0 mm; 5) Diffusion Tensor Imaging: FOV 240, 55 slices, 2.5/0 thickness, TR = 8700ms, TE = 122ms, Resolution 96 x 96, B values 0 and 1000, 30 directions and bandwidth 1408; 6) Arterial Spin Labelling: FOV 192, 14 slices, 6/1.5 thickness, TR = 2500ms, Te = 11ms, Resolution 64 x 64, TI (1) = 700ms, TI (2) = 1800ms, Bandwidth 2232 and 101 measurements; and 7) Susceptibility weighted imaging (SWI); 1 x 40 slice axial volume, 4mm thickness, 0.8mm gap, in plane resolution of 0.5 x 0.5 mm (TR = 40ms, TE = 30ms, flip angle = 15°).

##### Visual assessment of white matter disease progression and medial temporal lobe atrophy

White matter hyperintensities (WMH) at baseline will be rated using the Scheltens scale (range, 0 to 84 points) [62]. Visual assessment of progressive white matter disease will be performed using the modified Rotterdam Progression Scale [63]. This score ranges from 0 to 9, scoring stable (0) or increase (1) in 9 regions. Intra and inter rater reliability for these methods will be assessed and have previously been shown to be high [64]. Medial Temporal lobe Atrophy (MTA) has been linked to both progressive white matter disease [65] and cognitive decline [66] in MCI participants. Visual assessment of the medial temporal lobes will be performed on coronal T1 images from the volume set according to the 5 point (0–4) Scheltens scale from the average score of the left and right sides [67].

##### Volumetric MRI analysis

Volumetric analysis of the white matter lesions will be performed at a laboratory equipped with FSL (http://www.fmrib.ox.ac.uk/fsl) and Analyze (http://www.analyzedirect.com) software packages. A semi-automated threshold region of interest (ROI) based approach to white matter lesions will be used. All ROIs selected will be reviewed by a neuro-radiologist to confirm accurate identification of lesions and accurate identification of contours of lesions. The volume of the lesions will be summed to give a total lesion volume or total WMH. This will be standardized to the whole brain volume to control for individual differences in brain size. A fully automated measure of white matter lesions will be undertaken using published automated image processing algorithms [68] and compared with the results of the semi-automated supervised technique. Similarly, volumetric analysis of the medial temporal lobe will be performed manually and in an automated fashion. The manual segmentation will be performed by identifying the contours of the hippocampus on coronal T1 weighted images, employing well defined anatomical landmarks [69]. This will be compared to an automated technique. Hippocampal volumes will be standardized to the whole brain volume to control for individual differences in brain size.

##### PET scan

The PET ligand F-18 Florbetapir (previously known as F-18 AV-45) will be used to image in vivo amyloid, based on the presence of amyloid plaques in the brains of AD patients. This compound was conceived of, synthesised, and clinically developed at the University of Pennsylvania and Avid Radiopharmaceuticals Pty Ltd (Philadelphia). This drug has completed Phase III clinical trials and been submitted to the Food and Drug Administration for marketing approval as a diagnostic test for the detection of amyloid plaques. No adverse effects have been found during the clinical development of this compound.

##### PET image acquisition

Participant preparation consists of intravenous (IV) catheterization and immobilization of the head with a Velcro strap. The scan will require an IV bolus administration over 30 sec of 370 MBq (less than 5 micrograms) of high specific activity F-18 Florbetapir. Fifty minutes post injection of the compound, a 15 minute scan will be acquired using a Phillips Allegro PET camera.

Co-registration of each participant’s MRI with the PET images will be performed and an MRI ROI template transferred to the co-registered PET. Standardized uptake value ratios (SUVR) will be generated by normalizing to the cerebellar cortex. Neocortical beta-amyloid burden will be expressed as the average SUVR of the area-weighted mean of frontal, superior parietal, lateral temporal, lateral occipital, and anterior and posterior cingulate regions.

#### Randomization and blinding

Randomization will be undertaken in blocks of six participants (three in each of the treatment arms). The blocks were generated in STATA 10 (StataCorp, TX, USA). An investigator not directly involved in the recruitment or assessment of participants will perform allocation to study groups. Due to logistic difficulties, participants will not be blind to the intervention and sham PA will not be used. However, the “clinical staff” involved in the collection of endpoints will not be aware of group allocation (single blind). Blinding will be supported by the performance of cognitive and physical assessments at different locations and explicit instructions to participants and research staff not to discuss issues related to PA during the assessments.

#### Intervention

The intervention period will be 24 months divided into 4 stages; Stage 1: 0–6 months; Stage 2: 6–12 months; Stage 3: 12–18 months; Stage 4: 18–24 months, with review at the end of each stage (Figure 1). The intervention will comprise three components: the PA program, the behavioral intervention package, and telephone monitoring. Participants randomized to the intervention will return for a PA workshop within two to four weeks of their baseline visit. During this 60-minute session, the PA RA will give participants their program manual and explain the participants’ PA program and the behavioral intervention package.

#### Physical activity program

Participants will be advised to perform at least 150 min/week of moderate PA as per the PA recommendations for older people [70]. Where walking is a suitable and acceptable option to the participant, this will be a primary PA recommendation. Examples will be given on how this can be achieved. The program will be individualized based on the CHAMPS and SCI data, and the person’s interests. Activities prescribed will take into account health problems or other limitations, and participants will be instructed to start slowly and progress gradually taking eight weeks to reach the target amount and intensity. Participants will be encouraged to achieve the 150 min/week by completing 3 x 50 minutes sessions (most PA classes range between 45–60 minutes). In previous studies with middle-aged and older women using this format, we have demonstrated good retention, adherence and improvements in cardiovascular fitness and blood pressure [71]. Participants who already perform 150min/week of PA will be encouraged to increase their PA by adding one 50-minute session. Some participants might choose to complete activities in community centers. This home-based PA program was successful in maintaining retention, increasing PA and improving cognition in participants with MCI and those with subjective memory complaints [24]. Modifications have been made to the PA program and other components of the intervention to enhance motivation and to maintain PA over the 24-month intervention.

In order to maintain standardization of the physical activity programs one CI will monitor the programs prescribed by the PA RA. The PA program will include instructions on how to read the program, complete the activities, record their sessions, and exercise safely. Participants will be given a simple diary to record their PA. The diaries, once completed, will be mailed back to the PA RA at the end of each month.

Adherence during the intervention will be calculated from the number of sessions completed and recorded on the exercise diaries. This will be expressed as a percentage of the number of sessions completed relative to the number of sessions prescribed.

#### Behavioral intervention

Participants will receive the same educational material and recommendations for a healthy lifestyle as the control group. In addition they will receive a manual containing the PA program and the behavioral intervention (BI). The intervention program will be based on the Stages of Change model modified for PA [58], which has been shown to be effective in increasing and maintaining PA in middle-aged and older women [71]. This approach is based on the development of self-efficacy (the belief that one has the ability to perform a task) to promote change [72]. It will also draw on another key component of social cognitive theory and behavior change self-regulation; the personal regulation of goal directed behavior or performance [72,73]. The BI program will aim to develop self-efficacy by promoting practical strategies to enhance both physical skills and self-regulation skills. We will use our previously successful strategies [71] with the addition of greater emphasis on identifying and setting goals, self-monitoring, giving relevant feedback, review of progress and identifying action steps to enhance self-regulation skills.

Strategies to increase adherence to the program will be discussed during the workshop and worksheets will be included in a manual. During the 24-month trial, the PA RA will mail newsletters at regular intervals to participants, which contain additional motivational information. This will also be reinforced during the 18 telephone calls made at regular intervals. Participants will be contacted by telephone for a standardized and structured 15-minute interview to monitor and give feedback on their progress and encourage their continuing adherence. In addition pedometers will be given to the participants for the duration of the study and midway through each 6-month period they will be asked to record their daily steps for 4 weeks while trying to reach a personalized target. Pedometer-based interventions have been shown to be an effective way to help maintain PA in older adults [74]. Participants will also be given a PA report after their follow-up assessments. Giving feedback about progress and increasing participants’ perceived benefits of being more physically active has been shown to increase program adherence [75].

#### Content and program evaluation

At the end of each follow-up visit, the intervention group participants will be asked to complete a brief interview questionnaire on the content and processes of the program including how easy the program was to understand and follow, and any barriers that were encountered.

#### Control group

Control group (usual care) participants will receive educational material and recommendations for a healthy lifestyle (other than PA). Participants will be contacted by telephone at the same frequency as the intervention group to ensure that the control and intervention group have similar treatment except for the actual intervention. Conversation for this group will be limited to their general health and will not include discussion about PA. At the end of each follow-up visit, control group participants will be asked to complete a brief interview questionnaire related to their involvement in the study. At the conclusion of the study, these participants will be offered the opportunity to attend an educational session on PA.

### Statistical methods

Participants who drop out during the trial will be invited to return for the follow-up assessments. We will use imputation by chain equations (ICE) to estimate possible missing outcomes in an intention-to-treat analysis (primary analysis). A complete-case analysis will also be conducted, which includes participants with valid data at all time points. We will use multilevel regression models to take into account repeated measures and intra-individual variability (mixed models). These models will be adjusted for confounding should the randomization produce unbalanced groups.

The volumetric change in WMH white matter load at 24 months and the modified WMH change score will be assessed using an independent 2-sample *t*-test or Mann–Whitney *U* test, depending on whether the data satisfies assumptions of normality. The modified WMH change score may also be collapsed into a smaller number of categories and assessed using a chi-square test. General linear models will then be used to estimate treatment effects after correcting for initial WMH severity, age, depression, MTA, cognition and VRF. The predictive contribution of these confounding variables will also be investigated. Model assumptions of normality and constant variance will be checked and data will be transformed if required. A multiple imputation method will be applied to replace missing observation values during the follow-up for key analysis variables if necessary. The change in secondary outcomes, cognition, fitness, depression, quality of life and functional levels, between baseline and 24 months and between groups will be analyzed. General linear models will be fitted for each outcome separately and known confounding variables will also be included in this analysis. Frequency counts and chi square analyses will be used to determine the distribution of subjects for retention in the study and demographic data. Changes in PA, fitness, blood pressure, body weight, body composition and socio-psychological variables will be analyzed across the entire follow-up period, using repeated measures mixed model analysis. Amyloid status is another important explanatory variable in the progression of WMH. This will be incorporated into the general linear model described in the analysis of the primary outcome. The interaction effect between amyloid status and treatment will also be included in the model to assess whether amyloid status influences patient response to treatment.

#### Sample size and power calculation

The primary outcome of interest in this study is change of WMH on follow-up MRI after two years. To our knowledge there is no study having investigated the effect of PA on progression of WMH in older adults with SMC and MCI. However in a recent RCT, Richard et al. [76] compared WMH progression in 123 participants with AD who received either vascular care or standard care over a two-year period. Vascular care was defined as treating vascular risk factors as good as possible. Scans were analyzed and WMH at baseline were rated using visual semi-quantitative rating scales. Progression of WMH was measured using the modified while matter lesion (WML) change score (range 0 to 9) [63]. After two years participants in the vascular care group had less progression of WMH compared to the standard care group (1.4 versus 2.3, p = 0.03) with a significant linear trend (p=0.009). We hypothesize that in our participants the effect of the PA program on WMH would be similar to that of vascular care intervention. Based on the WML change, with a medium effect size of 0.5, 80% statistical power and a two-sided alpha error level of 0.05, we therefore would need 65 participants per group. Allowing for 15% loss to follow-up we will need 76.5 participants per group. We will recruit 78 participants per group for a total of 156 participants.

## Discussion

With the global incidence of dementia projected to rise dramatically over the next several decades, developing effective strategies to reduce the risk of cognitive decline or slow its progression is critical. Physical activity has been successfully used to modify VRF [77] and it has more recently been proven to benefit cognition in individuals with MCI [22,24,78]. Since white matter changes are considered a risk factor for cognitive decline, testing the efficacy of PA as a strategy to delay the progression of white matter changes in those at risk is timely.

This trial builds on the strengths of the Fitness for the Ageing Brain Study (FABS) [24] and, if successful, will provide proof of concept. In contrast to FABS and other RCTs on PA and cognition, AIBL Active investigates the effect of PA on biomarkers and progression of WMH in older adults with SMC and MCI and VRF. Building on the AIBL study, we will also be able to examine the associations between trial outcomes and brain beta-amyloid load measured with PET. This can help to identify the clinical populations that may benefit the most from such an intervention. The findings have the potential to inform practitioners and provide the impetus for translation into community programs. This trial may represent an affordable and safe method to delay the onset of cognitive decline and cognitive impairment in older adults at risk.

## Competing interests

The authors declare that they have no competing interests.

## Authors’ contributions

All authors are members of the AIBL Active research team and participated in the implementation of the study. The physical activity and behavioral intervention program was developed by KC. EC is the project coordinator and drafted the manuscript. NL, PD, DA, CS, OS, KE, PP, CM, CR, RM and KC conceived of the study, participated in its design and coordination and critically reviewed the manuscript. All authors read and approved the final manuscript.

## Pre-publication history

The pre-publication history for this paper can be accessed here:

http://www.biomedcentral.com/1471-244X/12/167/prepub
